# Factors influencing the scientific performance of Momentum grant holders: an evaluation of the first 117 research groups

**DOI:** 10.1007/s11192-018-2852-1

**Published:** 2018-07-20

**Authors:** Balázs Győrffy, Andrea Magda Nagy, Péter Herman, Ádám Török

**Affiliations:** 1 MTA TTK Lendület Cancer Biomarker Research Group, Institute of Enzymology, Hungarian Academy of Sciences, Magyar Tudósok körútja 2, Budapest, 1117 Hungary; 2 2nd Department of Pediatrics, Semmelweis University, Tűzoltó utca 7-9., Budapest, 1094 Hungary; 3 Department of Economics, University of Pannonia, Egyetem u. 10, Veszprém, 8200 Hungary; 4 Department of International Economics, University of Pannonia, Egyetem u. 10, Veszprém, 8200 Hungary; 5 Department of Economics, Budapest University of Technology and Economics, Magyar Tudósok körútja 4, Budapest, 1117 Hungary

**Keywords:** Scientific performance, Impact factor, Review, Publication activity, H-index, Momentum program

## Abstract

The Momentum program launched in 2009 provides funding of up to 1 million Euro to establish new, independent research groups at Hungarian academic institutions. Here, our aim was to determine factors associated with the scientific output of these research groups. Publication data were downloaded from the Hungarian Scientific Work Archive (www.mtmt.hu), impact factor data were obtained from Thomson Reuters (jcr.incites.thomsonreuters.com), and journal ranks were extracted from the Scimago Journal Rank database (www.scimagojr.com). Investigated input features for each grant holder include gender, degree, targeted category, international mobility, international grants, number of publications, total number of citations, H-index, best publications, impact factors in the last 2 years, and assessment scores provided by the experts. Evaluated performance indicators include cumulative impact factor, number of D1 publications, and number of first/last author D1 publications during the grant running time. Grant holders’ publication output increased by 23 and 52% for life sciences and material sciences researchers. Scientific performance was independent from gender, degree, international grants, category applied for, and citations received for the best pre-grant publication. Those with international mobility had significantly lower scientific output (yearly impact factor, number of D1 publications, number of first/last author publications). Scores received from grant review experts were independent from later publication activity. The strongest correlations were observed between scientific output and total number of citations, H-index, and impact factor in the last 2 years pre-grant. In summary, group leaders with a dynamic publication track record were able to attain the most additional momentum. Our results can help accelerate and improve future grant review processes.

## Introduction

The purpose of this study is to provide new ideas for researchers interested in grant evaluation efficiency related issues. More precisely, the authors intend to shed some light on the critical points of project selection based on *ex ante* grant evaluation. A cautious question is asked about the overall usefulness of techniques applied in such a selection. Our research hypothesis is similar to the Antitrust Paradox in a certain way (Bork 1993), a classic in the literature of competition economics. In this Paradox, Bork states that antitrust policies expected to raise consumer welfare sometimes merely reduce it due to high institutional costs and efficiency problems. This Paradox has much relevance for a wider range of regulatory policies, including the selection of scientific projects.

Using the example of a prestigious R&D funding scheme used in Hungary, we argue that certain project selection techniques do not necessarily award applicants with the best research/publication potential. Efficiency problems of grant selection processes are brought to the forefront by our performance analysis of projects concluded within the Momentum (Lendület) Grant Scheme (MP). This research is partly justified by the need for a performance assessment of MP including more effective tools of *ex ante* and *ex post* grant evaluation.

MP was launched in 2009, with an annual award budget of up to EUR 1 million. This budget has been financed from resources provided by the government to the Hungarian Academy of Sciences (HAS), a non-governmental public organization, administering MP along with a string of other R&D funding schemes. MP’s innovative character is based mainly on the fact that its priority target is to provide an effective incentive to Hungarian researchers working abroad to return to their home country. Therefore, Momentum is meant to offer internationally competitive financial conditions to its grantees. Grants are awarded for 5 years, with new applications considered once each year.

MP’s grant eligibility criteria include a PhD degree from an internationally renowned university, and documented research or teaching experience obtained in internationally prominent research centers of the field. Furthermore, continuous publication activity in leading scientific journals of the field based on original research papers, and a good record of invited speakership at scientific conferences are also requested along with suitable grant-attaining performance. These criteria are in line with international standards of grant eligibility.

Momentum does not offer only lucrative financial conditions to its grantees. It also sets truly international standards of scientific performance. This study is the first comprehensive analysis of Momentum-related research performance. Our intent was to base this analysis on a multi-dimensional set of indicators of research output in line with the most recent scientometric standards.

Simple and basic bibliometric indicators include:

- total number of publications,
- total number of citations,
- total number of publications with at least a number of citations,
- total number of citations for the most cited papers,
- average number of citations per paper (ACPP),
- H-index (Hirsch 2005; Hou et al. 2018; Bornmann and Leydesdorff 2018)

and are used to measure the influence and citation impact of journals or the scientific performance of researchers.

Several bibliometric indicators have been developed to measure the productivity of a researcher and of his or her research (Bornmann et al. 2017; Nabout et al. 2018). A number of authors have provided thoughtful reviews of the limitations of bibliometric analyses (Texeira da Silva and Dobránszki 2018; Costas and Franssen 2018). These limitations include biases of bibliometric comparisons across fields or countries (Gingras and Khelfaoui 2018); a tendency to favor older researchers with more experience (Kwiek 2018) or wider networks (Ronda-Pupo and Katz 2018; Akbaritabar et al. 2018); political motivations (Kostoff 1998); and a susceptibility to bias, such as self-citations (Thelwall 2018; Drew et al. 2016). The as yet unresolved problems of multiple co-authorship (Bao and Zhai 2017; Ronda-Pupo and Katz 2018), and the fact that the annual publication capacity of top journals measured in pages varies widely across fields of science could also be mentioned in this respect. These problems make a comparison of publication performance between fields of science unreliable to a certain extent.

Bibliometric indicators used as researcher assessment tools can affect the behavior of researchers seeking better positions, tenure (Picinin et al. 2016; Hoppen and Vanz 2016; Walters 2016), and even research grants. This has been a recurrent concern in academia, because of the bias that it may introduce in the evaluation of research funding applications or promotions (Yan et al. 2018). Despite a string of caveats, however, bibliometrics still are used widely as an evaluation tool to assist selection processes in R&D funding (Gunashekar et al. 2017).

The main reason for this seems to be the strong need for a more or less objective measure to compare grant applications. Reviewers of grant applications have to be accountable on the results of the selection process both to the funding agencies and, ultimately, to the scientific community, including the applicants. This accountability may be supported strongly by adequate methods of grant proposal evaluation.

### Evaluation of research grant proposals

Researchers “are forced into a competitive environment driven by evaluation for the allocation of these scarce funds” (Heinze 2008). The rationale for this competition-based funding policy is the hope that competition might help bring forward the best ideas and research topics. The assumption underlying MP is similar: a funding scheme in Hungarian research with amounts of grants (“payoffs”) comparable to those in Western Europe/United States is likely to create heated domestic competition for funds, which could attract “players from top leagues”.

Success in obtaining research funding is also an important factor in researchers’ performance evaluations. Funding agencies have great influence on research directions, topics, and formats. Zhao et al. analyzed the relationship between funding and the usage count of the Web of Science database. A positive correlation was found between usage and research funding (Heinze 2008; Zhao et al. 2018).

Lewison and Dawson were among the earliest to investigate the link between grant-funding bodies and procedures and the research they supported (Lewison and Dawson 1998). They found that the count of funding acknowledgements of a paper generally was a predictor of higher impact and therefore a desirable outcome. The association occurred because research with more funding obtained more positive peer reviews and was therefore more likely to be of higher quality. A higher number of funding bodies correlated with research published in journals of higher citation impacts. Journal articles from G9 countries were analyzed by Huang and Huang (2018) to identify the distribution of funding agencies and research funding. They found that China has the highest proportion, while Italy the lowest proportion, of funded articles based on scientific output. The relationship between research funding and bibliometric indicators has also been analyzed (Zhao et al. 2018).

Awarding panels for major funding schemes by the National Institute for Health Research (NIHR) in the United Kingdom have been analyzed recently. It was noted that the use of bibliometrics is used primarily in the initial individual assessment of candidates (Gunashekar et al. 2017).

The distribution inequality of research funding for projects of the Chinese Academy of Sciences have been analyzed by Li et al. It was found that although there is evidence of the overconcentration of research funds, inequality decreased over the period that was analyzed (2011–2015). Delimiting the number of projects to which one researcher can apply at one time and promoting collaboration between researchers through project subdivisions has been suggested (Li et al. 2017).

Mejia and Kajikawa used bibliometric tools to examine whether funding agencies allocate resources to innovative research rather than mature fields. Analyzing the Acknowledgements sections of sponsored articles, European funds were found to be among the most recognized sponsors of change maker technologies, while Japanese agencies and the NIH focus more on incremental research (Mejia and Kajikawa 2018). The choice of R&D funding schemes by policymakers (Lee and Bozeman 2005) should strive to keep a balance among the different forms of funding instruments applied, while still ensuring high scientific quality. Rigby compared the scientific quality of 1010 scientific papers from the ISI (Institute for Scientific Information) database produced under two contrasting forms of funding instruments: single grant holders and collaborative networks within a research system in the Austrian science system (Rigby 2009). Interestingly, he found no statistically significant difference in the ArcSinh transformed citation counts of papers from the two main forms of funding for basic science. This may suggest that funding agencies and researchers have succeeded in ensuring that different research instruments nevertheless are conducive to similar levels of scientific excellence.

According to research on Japanese academia (Shibayama 2011), large-size grants have become more common, and multiple awarding (one researcher receives more than one grant simultaneously) has become more frequent. Funding distribution was found more unequal than the distribution of publications as an output indicator. Considering substantial disparities in researchers’ performance (Lotka 1926), performance-based funding inevitably results in highly unequal distribution (Shibayama 2011). This result raises concerns regarding the degree of necessity or relevance of performance oriented expert reviews of grant applications.

Ridby and Julian show that research has a greater citation impact when certain pre-conditions for “double dipping” are met (for example, when funding comes from more than one organization or the organizations fund someone’s research in very similar areas) (Rigby and Julian 2014). They analysed projects funded simultaneously by EMBO (European Molecular Biology Organisation) and HFSP (Human Frontier Science Program). They concluded that authors achieved a higher average citation count for their papers when they were funded by both agencies at the same time. This finding also may suggest that attempts to limit the number of grants simultaneously received might in fact compromise the production of more frequently cited papers (Rigby and Julian 2014).

Reinhart examined the peer review procedure of a national science funding organization (Swiss National Science Foundation) by means of the three most frequently applied criteria, reliability, fairness, and validity (Reinhart 2009). Bibliometric analysis provided evidence that the decisions of a public funding organization on basic project-based research are in line with the future publication success of applicants. The paper’s argument is in favor of a broader scope of approaches and methodologies applied in peer review research, including, for example, content analysis (Reinhart 2009).

A timely analysis of participation in the 8th European Framework Programme for Research and Innovation (EU FP) Horizon 2020 by Norway was investigated by Enger and Castellacci (2016). Their dataset comprises the entire population of research organizations in Norway, enabling them to distinguish between non-applicants, non-successful applicants, and successful participants. Their econometric results indicate that the propensity to apply is enhanced by prior participation in EU FPs and the existence of complementary national funding schemes; furthermore, the probability of succeeding is strengthened by prior participation as well as the scientific reputation of the applicant organization (Enger and Castellacci 2016).

### Peer review

The peer-review process often is used for assessing scientific output (see for example García et al. 2018; Kovanis et al. 2017), academic promotions, or PhD theses (Ortega 2017). Therefore, it can greatly influence knowledge production and knowledge distribution and is also crucial to a scholar’s career advancement (Jirschitzka et al. 2017). Grants rarely are awarded without it (Lundberg 2002).

Studies of peer-review processes are numerous regarding the methods used and the factors identified as having an influence on the process (Batagelj et al. 2017; Rigby et al. 2018). The monitoring of its effectiveness and its influence upon research productivity has been the subject of great interest (Horlesberger et al. 2013; Bruce et al. 2016). Its objectiveness has been debated by many, as has its effect on scientific performance (Casnici et al. 2017). It also may be the subject of inconsistencies that are difficult to remediate (Haug 2015). Other complaints regarding the peer-review process include the reviewers’ errors (Zucker 2008), limited time spent on review (Rocha 2001; Perrin 2008), profligacy of academic time (Smith 1997), and geographic and language bias (Burns and Fox 2017). Truly innovative research projects might be hindered in both publication and funding by the review process (Spier 2002).

The main difference between peer-reviewing publications and research grants is that author(s) intend to announce research results when publishing, but applications for research funding are only tentative projects (Fang 2011). The question arises of how a peer-review process of a grant could predict a result of a research project that is still at its starting point, and this is also one of our main research questions regarding Momentum. In the present study, our goal is to determine factors associated with the scientific output of Momentum research groups.

## Methods

### Publication data

We took advantage of the Hungarian Scientific Work Archive (*Magyar Tudományos Művek Tára*—www.mtmt.hu) to assess all grant holders (including current grantees and those already finished their 5-year grants). The data were used relative to the grant running time (e.g., before and after starting the grant was set by using a different year as a cutoff for each grant holder). Most grant holders update their database at the end of their grant’s yearly reporting period, which generally is in July. Bibliometric data were downloaded for all papers published in scientific journals for each grant holder. Conference proceedings, correspondence to the editors, and other non-peer-reviewed publications were omitted. Data were collected in November 2017.

IF data were obtained for each journal from Thomson Reuters (jcr.incites.thomsonreuters.com), and journal ranks were extracted from the publicly available Scimago Journal Rank database (www.scimagojr.com). SCImago is a research group dedicated to information analysis from the Consejo Superior de Investigaciones Científicas (CSIC), University of Granada, Extremadura, Carlos III (Madrid) and Alcalá de Henares (Spain). The SCImago page ranking was developed using the Google PageRank™ algorithm and includes 34,100 titles from more than 5000 publishers. A journal was assigned to be in D1 (first decile) if it ranked within the first 10% of journals within its subject area. For example, if one category has 50 journals, the first five of these were allocated into the D1 category. Notably, the D1 category was not established for the present paper but has widespread use by grant agencies in Hungary.

Performance indicators evaluated for each grant recipient include cumulative impact factor (IF), number of D1 publications, and number of first/last author D1 publications during grant running time and up to 3 years before the grant submission cutoff. Impact factor was used, as it is the most common measure also listed on the homepage of most journals. D1 was used because it enables the comparison of different scientific disciplines.

### Momentum grant holders’ characteristics

The above publication output features after receiving the grant were compared to the grant holder’s characteristics at the time of application. For this, a series of questions regarding the scientific qualities of the applicants were collected from their grant applications. These include degree, international grants, number of publications, total number of citations, H-index, citation data of the best publication, and impact factor in the last 2 years. Gender was determined by examining the name of the applicants. Since 2011, the Momentum grant scheme has had two distinct categories:”Momentum I” for early-stage researchers between 30 and 38 years old and “Momentum II” for experienced researchers between 39 and 45 years old. Data for these categories were collected from the online application system. A Momentum grant can only be executed in Hungary, and grant holders who moved to Hungary to execute the grant program were designated as fellows with “international mobility”.

Each Momentum grant proposal is evaluated by 3–4 experts. These experts provide a written description as well as a percentage estimation of the applicant’s performance. The percentage estimation can be in the range between 0 and 100. The reviewers’ scores for each grant holder were provided by the Momentum grant administration office.

### Statistical methods

Grant holders are assigned to three disciplines at the time of application – life sciences, material sciences, and humanities. Grant holders in humanities were excluded from our analysis due to markedly different publication characteristics. In humanities, many publications, such as books, do not have an impact factor and/or cannot be assigned into categories like D1. In particular, the mean number of publications was 33.3 in life sciences, 28.9 in material sciences and 11.4 in humanities, and 87.6, 90.7 and 30.1% of these publications (respectively, for the three disciplines) had an impact factor. An exception was psychology (mean number of publications of 37, and 97.3% of these had an impact factor): grant holders in this area were added to the life sciences discipline. Statistical analysis was performed for the two major disciplines separately. Scientific characteristics of the grant holders at the time of application and publication track record after obtaining the grant were compared by Mann–Whitney and Kruskall–Wallis tests and Spearman rank correlation. The threshold for statistical significance was set at *p* < 0.05 in each analysis.

## Results

### Overall performance indicators

**Fig. 1:**
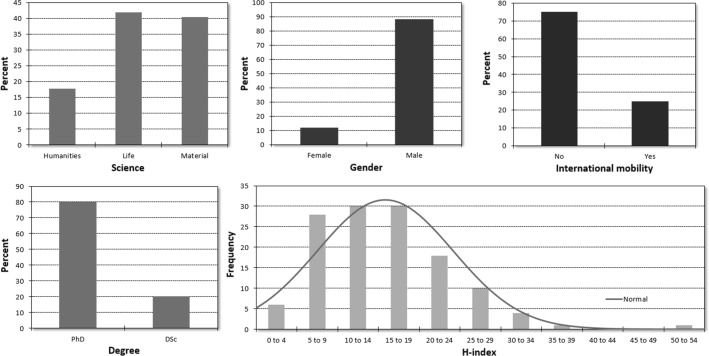
Descriptive characteristics of Momentum grants awarded in life sciences and material sciences (*n* = 117) including scientific discipline, gender, degree, international mobility, and H-index of the grantee at the time of application

A total of 117 research groups were evaluated. Descriptive characteristics of awarded Momentum grants are summarized in Fig. 1. Only grants already completed were included when assessing overall performance. An average grant holder in life sciences had 11.3 D1 publications and a yearly impact factor of 21.8. The mean number of D1 publications in material sciences was 10.7, and the yearly impact factor was 23.6. Almost half of the D1 papers were first/last author publications in both life sciences and material sciences (5.3 and 4.8, respectively). Aggregate publication indicators during the Momentum grant time are summarized in Table 1. A boost of 123 and 146% was observed in life sciences and material sciences, respectively, when comparing the mean of the cumulative impact factor in the first three complete years after grant award to the 3 years before the starting year of the grant. The boost for the number of D1 publications was also computed for the same period: the increase was 9 and 15% in life sciences and material sciences, respectively (in life sciences: 4.05 vs. 4.41; in material sciences: 3.73 vs. 4.28). Finally, when comparing the number of first/last author D1 publications between the last 3 years before and first 3 years after, an increase of 4 and 13% was observed in life sciences and material sciences, respectively (in life sciences: 1.65 vs. 1.73; in material sciences: 1.91 vs. 2.16).

**Table 1:** Aggregate performance indicators for life science (*n* = 60, A) and material sciences (*n* = 57, B) Momentum grants

	IF/year	Total IF	D1 publications	First/last author D1 publication	Boost (%)
A					
Min	3.6	21.4	1	0	13
Median	18.2	109.5	9.5	5	108
Mean	21.8	130.9	11.3	5.3	123
Max	60.5	363.1	41	19	350
B					
Min	0	12.78	1	0	21
Median	19.1	114.6	8	4	123
Mean	23.6	142.6	10.7	4.8	146
Max	83.1	498.7	51	16	471

D1 corresponds to journals within the top 10% of their respective categories. Boost is defined as the percentage ratio of the cumulative impact factors in the first 3 years of the grant divided by the impact factor of publications 3 years before obtaining the grant. The year of the award was excluded from this analysis as the grants generally start on the first of July. Total IF is the sum of the IF for all publications during the 5-year grant running time

### Characteristics with limited correlation to publication activity

Parameters without a significant correlation to publication efficiency are summarized first. Scientific performance was independent (no significant correlation) of gender in both life sciences and material sciences. In life sciences, the mean yearly impact factor of male grantees (*n* = 48) was 24.8 ± 5.9, and of female grantees (*n* = 6) 22.3 ± 14.3. The number of D1 publications were 10.9 ± 2.7 and 8.2 ± 3.9, and the number of first/last author D1 publications were 4.5 ± 1.2 and 3 ± 2.6 in male and female grantees, respectively. Female researchers had numerically higher yearly impact factors in material sciences (*n* = 44, IF/y = 23.2 ± 5.8 vs. *n* = 4, IF/y = 82.8 ± 202). Here, the number of D1 publications were 7.9 ± 2.6 and 1.5 ± 1.5, and the number of first/last author D1 publications were 4.0 ± 1.1 and 0.75 ± 1.5 in male and female grantees, respectively.

**Fig. 2:**
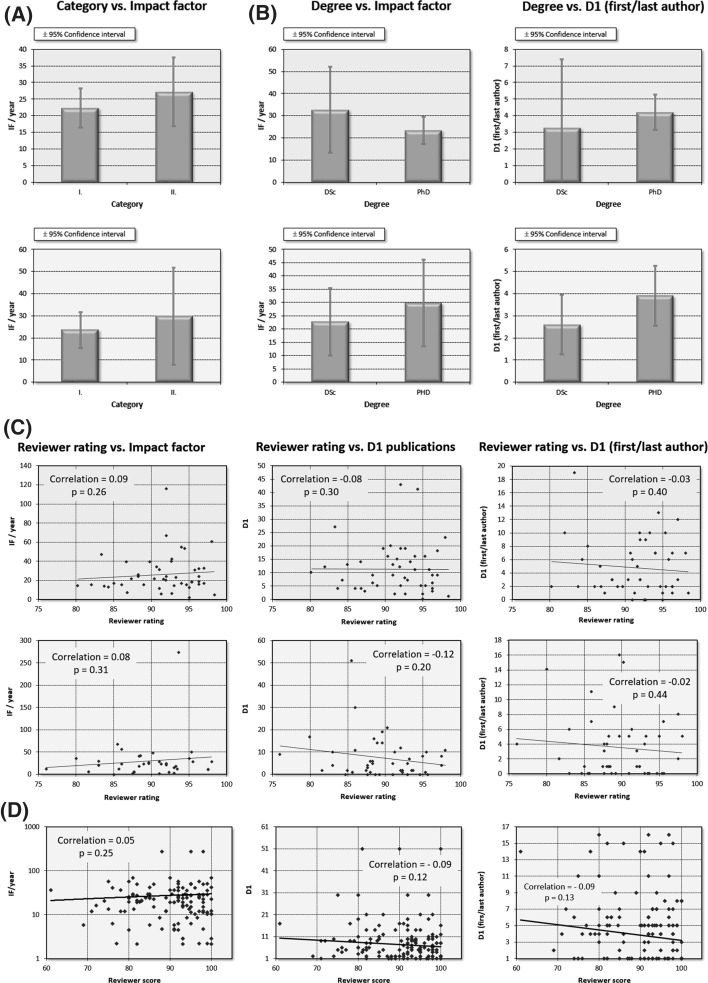
Selected parameters not correlated to publication performance in Momentum grant holders. Features include grant category (*n* = 99, **a**), degree of the applicant (*n* = 103, **b**), average ratings received from grant review experts (*n* = 94, **c**), and individual scores of each reviewer in material science grants (**d**). In each setting, data from life science grants have green background and data from material science grants have salmon background. D1: number of publications in journals within the top 10% of their respective category. DSc: doctor of the academy. IF: impact factor. None of the listed differences and correlations reach statistical significance. For **d** note that multiple dots will correspond to a single grantee

Two categories are available for a Momentum award, I and II, with a slightly higher budget for the Momentum II grants. When comparing these two Momentum categories, both achieved comparable publication records (in category I. *n* = 10, IF/y = 22.4 ± 5.8 versus category II. *n* = 25, IF/y = 27.2 ± 10 in life sciences and *n* = 17, IF/y = 23.5 ± 8 versus *n* = 25, IF/y = 30 ± 22 in material sciences). In both cases, those having Momentum II had higher output, but the differences were statistically not significant (see Fig. 2a). Of note, there was only a single category in the early years of the Momentum program, and therefore these grantees had to be excluded from this analysis.

We also compared the two different doctorate scientific degrees achievable in Hungary, PhD and DSc. Both degrees can be obtained by fulfilling the criteria set by a university (PhD) or the Academy of Sciences (DSc). By and large, a scientific performance of ten times that of a PhD degree is necessary to receive a DSc title, which is roughly equivalent to tenure in respected U.S. universities but does not offer employment. For example, if a given scientific discipline requires an IF of 6 for a PhD degree, then the requirement for a DSc degree is an IF of 60. In addition, only those who already mentored at least one PhD fellow are eligible for DSc. There was no significant difference when comparing the output of fellows with PhD and DSc degrees (in life sciences: IF/y = 33 ± 19 vs. IF/y = 23.3 ± 6, in material sciences IF/y = 19 ± 12 vs. IF/y = 29 ± 16, *p* = n.s., for DSc and PhD holders, respectively. See Fig. 2b). Similarly, the number of D1 publications and first/last author D1 publications was not different in life sciences (PhD: 9.8 ± 2.5, DSc: 14.6 ± 11.9 and PhD: 4.2 ± 1.0, DSc: 3.3 ± 4.1, respectively) and in material sciences (PhD: 6.8 ± 2.4, DSc: 5.7 ± 3.1 and PhD: 3.9 ± 1.4, DSc: 2.6 ± 1.5, respectively).

A primary focus of the Momentum program is to provide national support to researchers working in Hungary comparable to grants awarded by the European Research Council. In line with this, the applicants are also asked whether they have or had an ERC grant. In life sciences, the ERC grantees (*n* = 9) gained a yearly impact factor of 26.9 ± 7.5 and those without an ERC (*n* = 19) reached a yearly IF of 26.6 ± 13. In material sciences, only one candidate had an ERC with a yearly mean IF of 27.1, and those without an ERC (*n* = 20) published a yearly IF of 34 ± 27. Again, one must note that this information was only available for a fraction of the applications.

One of the criteria for a Momentum grant is to have highly cited papers in the CV of the applicant. Interestingly, when assessing the correlation between number of citations received for the best publication of the applicant and subsequent papers, there was no correlation in life sciences (*R*^2^ = 0.001). At the same time, a not-far-from-significant moderate positive correlation trend was observed in material sciences (*R*^2^ = 0.65).

### Grant review panel scores

Quite surprisingly, mean scores received from grant review experts show almost zero correlation to later publication activity (see Fig. 2c). In life sciences, evaluation of mean score and yearly impact factor, number of D1 publications, and number of first/last author D1 publications are not correlated (corr.coeff = 0.09, *p* = n.s.; corr.coeff. = −0.08, *p* = n.s., corr.coeff. = −0.03, *p* = n.s, respectively). Similar results were derived in material sciences (corr.coeff = 0.08, *p* = n.s.; corr.coeff. = −0.12, *p* = n.s., corr.coeff. = −0.02, *p* = n.s, respectively). Of note, reviewers who provided maximum scores for an applicant were excluded to avoid potential biases; the actual correlations were even smaller when including these.

Finally, when comparing individual scores received from each reviewer for each grantee, a trend towards a negative correlation was observed for score versus yearly impact factor, number of D1 publications, and number of first/last author D1 publications (corr.coeff. = −0.05, *p* = n.s.; corr.coeff. = −0.09, *p* = 0.12.; and corr.coeff. = −0.09, *p* = 0.13, respectively. See Fig. 2d).

### Factors correlated to publication activity in life sciences

**Fig. 3:**
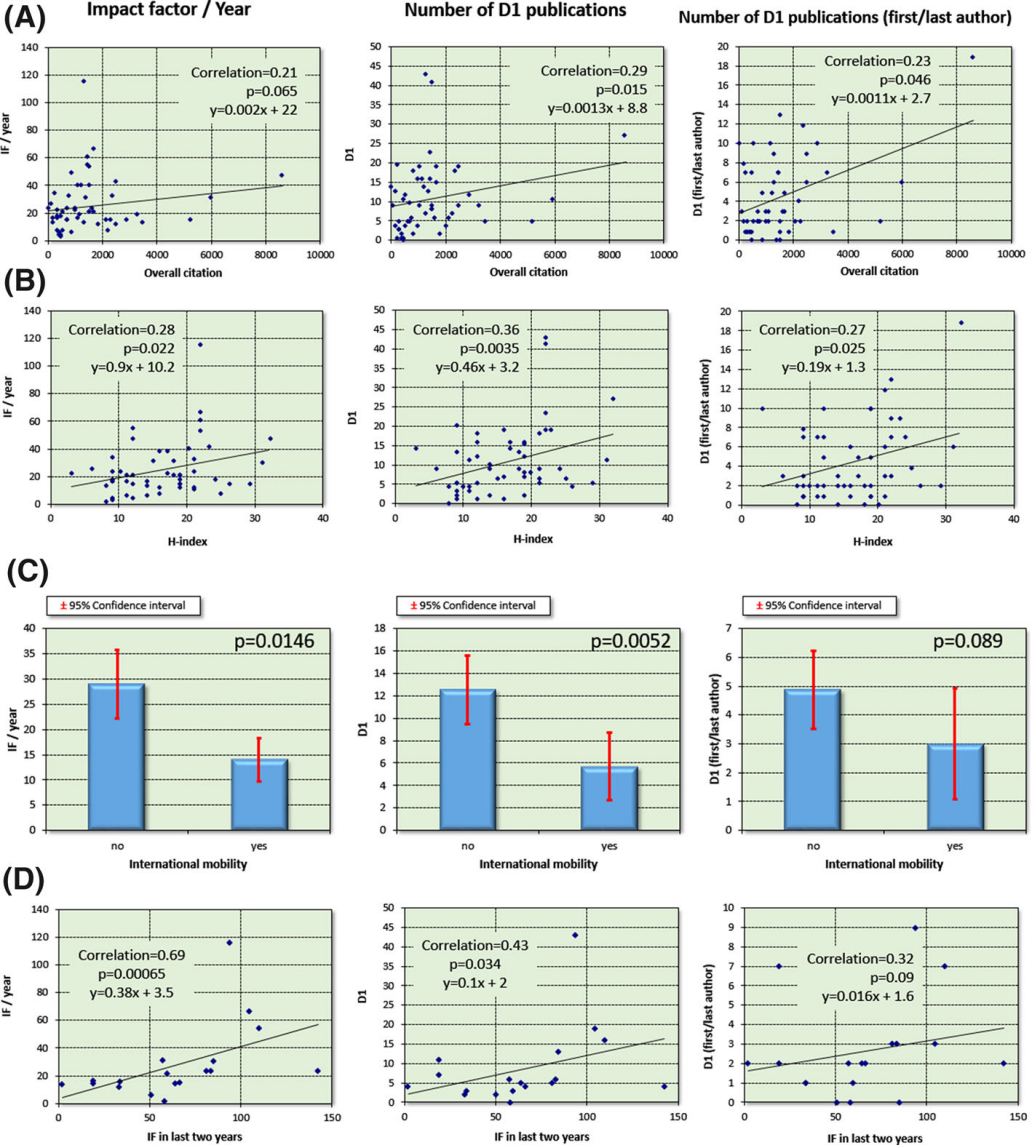
In life sciences, group leaders in a dynamically expanding tract record gain most additional momentum. Correlation between overall citation (*n* = 54, **a**), H-index (*n* = 55, **b**), international mobility (*n* = 52, **c**), and impact factor in the last 2 years before receiving the grant (**d**) and publication activity. D1: number of publications in journals within the top 10% of their respective category. IF: impact factor

Global metrics of the entire previous publication track record include total citation and H-index (see Fig. 3a, b). In life sciences, both of these show a positive correlation to yearly impact factor (corr.coeff. = 0.21, *p* = 0.065 and corr.coeff. = 0.28, *p* = 0.022 for overall citation and H-index, respectively), to number of D1 publication (corr.coeff. = 0.29, *p* = 0.015 and corr.coeff = 0.36, *p* = 0.0035, respectively) and to number for first/last author D1 publications (corr.coeff. = 0.23, *p* = 0.046 and corr.coeff. = 0.27, *p* = 0.025, respectively).

Similar to large international grants, the Momentum scheme facilitates both international and national mobility. A separate question in the grant application is set to identify those with international mobility. On the other hand, it was not possible to assess national mobility, because several applicants already held part-time positions or other significant ties with their respective host institutions. In addition, national mobility included movement within the same city in multiple cases. When evaluating international mobility, those who moved to Hungary from international/foreign research institutes after receiving the Momentum grant (*n* = 13) displayed significantly lower publication performance when compared to those already in Hungary (*n* = 39). These results were observed when considering yearly impact factors (14 ± 4.3 vs. 28.9 ± 6.8 for those with international mobility versus those without mobility, respectively), number of D1 publications (5.6 ± 3 vs. 12.5 ± 3), and number of first/last author D1 publications (3 ± 1.9 vs. 4.8 ± 1.4; see Fig. 3c).

Finally, the strongest correlation was observed between scientific output and impact factor of publications in the last 2 years before applying for a Momentum grant. Correlation was significant for yearly impact factor (corr.coeff. = 0.7, *p* = 6.5E−04) and number of D1 publications (corr.coeff. = 0.44, *p* = 0.034), and marginally significant for the number of first/last author D1 publications (corr.coeff. = 0.32, *p* = 0.09—see Fig. 3d).

### Factors correlated to publication activity in material sciences

**Fig. 4:**
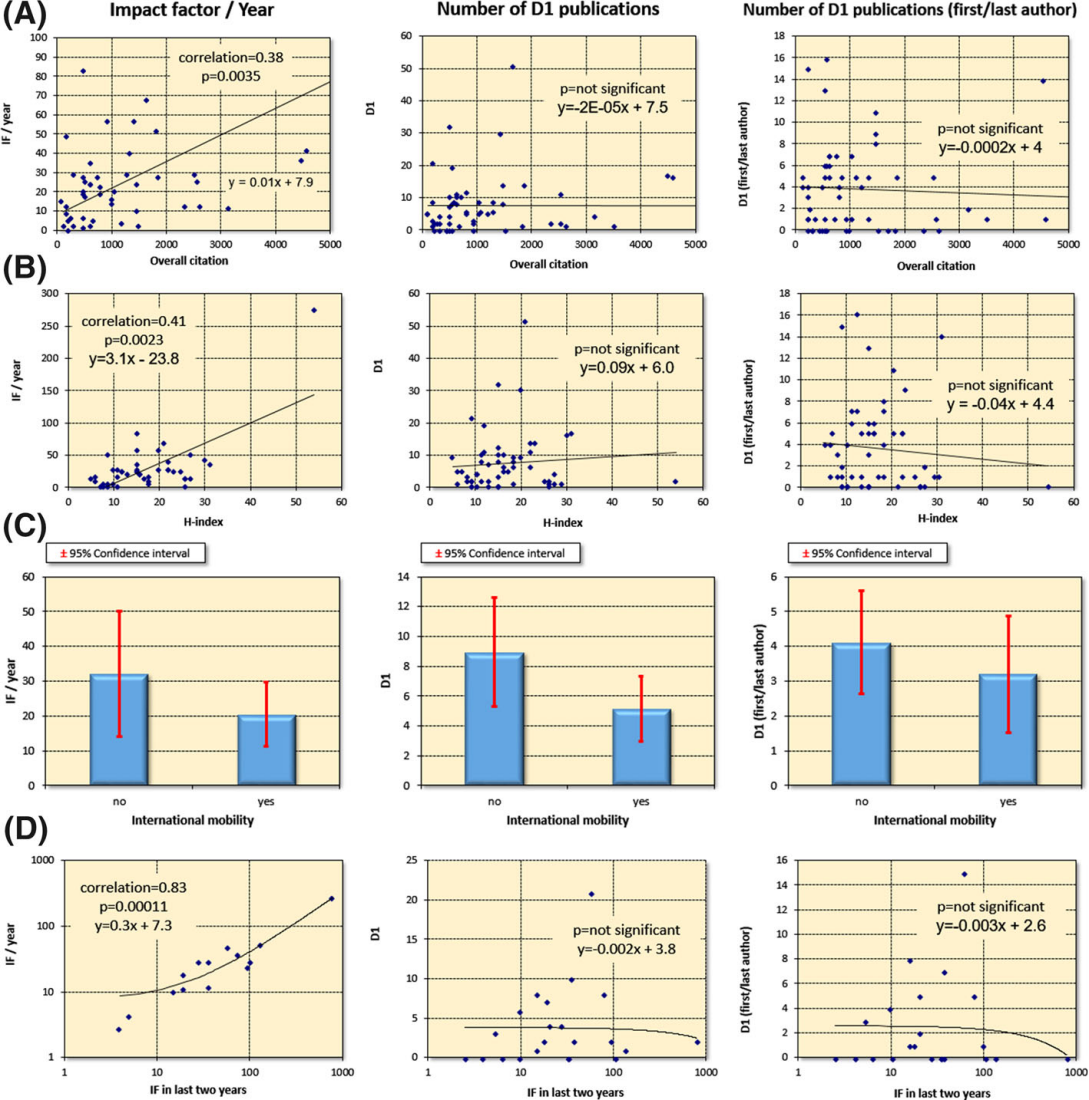
Trends in material sciences are similar to those in life sciences. Correlation between overall citation (*n* = 57, **a**), H-index (*n* = 55, **c**), international mobility (*n* = 30, **b**), and impact factor in the last 2 years before receiving the grant (**d**) and publication activity. Only the yearly impact factor reaches significance

Trends for overall citation, H-index, international mobility, and impact factor in the last 2 years were similar in material sciences. However, only yearly impact factor displayed statistically robust correlation, while the numbers of D1 publications and first/last author D1 publication were not significant (see Fig. 4a–d). In particular, yearly impact factor and overall citation (*R*^2^ = 0.73), and H-index (*R*^2^ = 0.44) show a strong positive correlation. As in life sciences, the strongest correlation was observed between impact factor in the last 2 years and yearly impact factor during the Momentum grant (corr.coeff = 0.83, *p* = 1.1E−04, see Fig. 4d).

## Discussion

For funding agencies such as the European Framework Programme, the European Research Council (ERC), or national funding agencies, a combination of bibliometric/scientometric and peer-review evaluation is needed when deciding upon funding research projects. We aimed to add new elements to diagnoses of peer-review systems used in the allocation of grants for research. Our main findings provide some potentially valuable input to policymakers interested in the design of efficient and effective research funding systems based on real scientific merit to the highest possible extent.

There is a natural consensus that research could not take place without funding. There remains, however, disagreement about the role of peer-review systems of funding agencies as influencers of the novelty and impact of research, and the extent of any interaction of funding provisions between different sources (Rigby and Julian 2014). One of the first comprehensive studies on the peer-review of funding agencies was by Cole et al. (1978), which dealt with the funding procedures of the U.S. National Science Foundation. Several studies analyzed in detail the research funding agencies in various countries and the measures taken by them to avoid potential biases (see for example, in Switzerland (Reinhart 2009) and in Sweden (Sandstrom and Hallsten 2008)). Some authors suggested a random distribution of funding (Geard and Noble 2010), a more careful selection of peer-reviewers (Marsh et al. 2008), person-directed funding (Azoulay et al. 2009), post hoc normalization of reviews, equal fund allocation (Bollen et al. 2017) or a method based on network flow theory (Cechlarova et al. 2014).

**Fig. 5:**
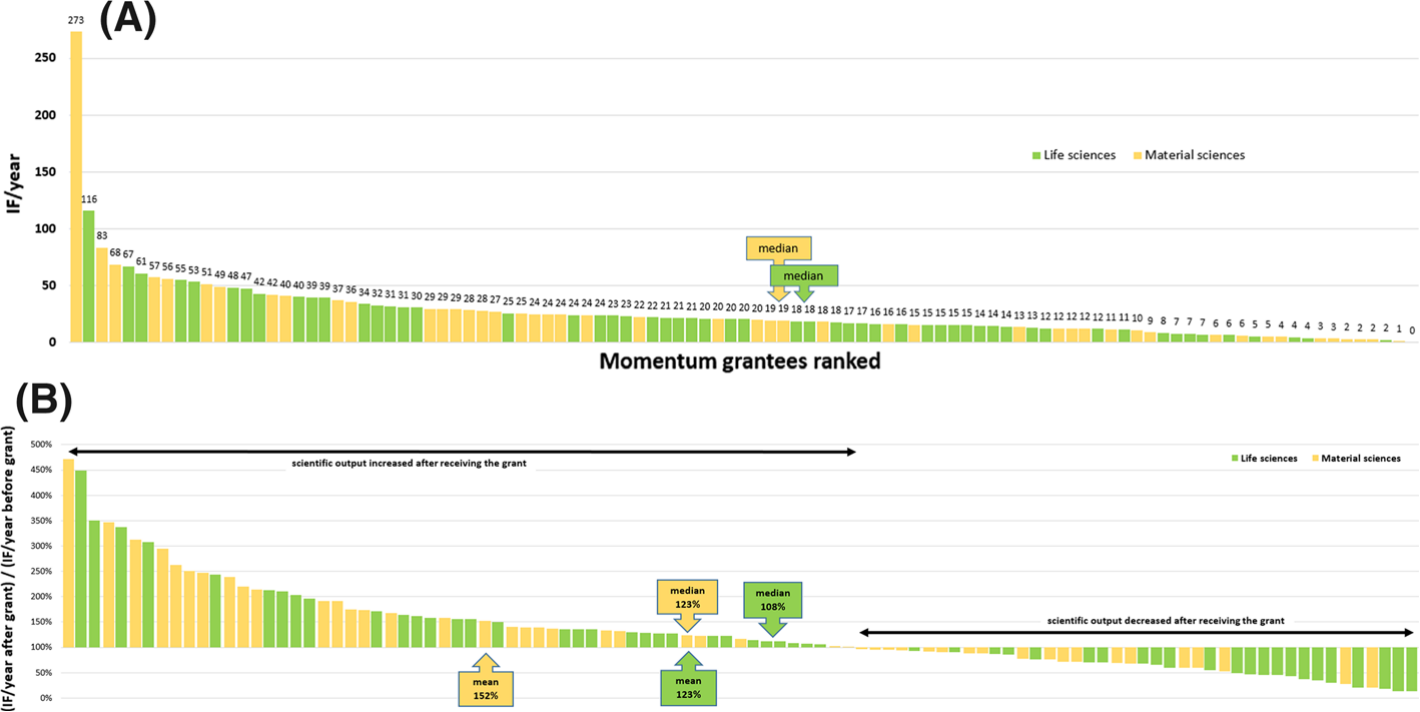
Ranked scientific performance of all grantees (*n* = 117). Yearly impact factor for all grantees with at least 2 years follow-up shows almost no difference between life sciences and material sciences (**a**). Ranked scientific output of all grantees after receiving the Momentum grant compared to output before grant award (**b**). In the **b** plot, 100% represents a performance identical before and after receiving the grant

A critical point shared by all such funding systems is selection-oriented peer review which can be regarded as a special kind of forecasting. A core challenge for this special forecasting activity is assessing future research performance on the basis of the quality of past research output. According to the present analysis, a selection from Momentum candidates/applicants has been successful in that the winners were able to improve their scientific output significantly during the grant period. In general, a considerable performance increase is observed after the establishment of a Momentum research group (see Fig. 5).

The picture becomes much more colored if a string of different performance indicators is used and compared. The message still remains mostly positive. Group leaders already in a dynamically expanding phase were able to attain the most additional momentum. Impact factor has became subject to intense discussion in recent years. Our analysis, however, confirms its quality and merit in certain types of prospective assessments of scientific performance. Namely, our results have shown that the impact factor of the last 2 years before the Momentum grant was the most significant predictor of scientific output in both life sciences and material sciences.

Despite of the surprisingly good performance of *certain* indicators in predicting scientific output, our overall impression of such predictions has a much more mixed character. We have found that, much more often than not, grant review experts failed in predicting future publication activity. Momentum grant applications are reviewed by 3–4 national and international experts. The experts give a percentage estimation between 0 and 100 for an application; typically, there are no winners with a mean score below 75%. Reviewer evaluation seems to be important, as ultimately this is the only factor actually considering the quality of the proposed research (all other factors evaluated here can be derived for any researcher even without a Momentum grant application).

Two remarks are in order at this point. First, peer reviews are not zero-cost at all. This means they consume time, human resources, and such money for which, at least in part, a much better use could be found in the funding scheme in question. Second, we do not suggest giving up the practice of pre-grant peer reviewing. We simply recommend a comparative cost–benefit analysis of pre-grant peer reviews, encompassing a wider array of research grant systems both in Hungary and on the international level.

Our final finding is related to the original, “strategic” purpose of the Momentum program. It had been launched based on the assumption that Hungarian researchers well established abroad but returning home as Momentum grant holders will affect research performance positively in Hungary. This expectation has materialized without any doubt, but a closer scrutiny of the performance of such “repatriated” scientists is not exempt from surprise. It has been shown that international mobility tends to reduce publication output in this special case. Thus, one might ask whether it is advisable at all to allocate funds to stimulate the return of such researchers instead of prioritizing the support for local researchers.

This conclusion may be relativized due to a relatively small sub-sample size for international mobility and the fact that the number of returning Momentum grant holders has decreased from the starting year of Momentum to this day. It is also true that the category of “returning” scientists includes a variety of research careers from tenured professors at a high-ranking university to associate professors at a less quoted teaching institution.

Nevertheless, this apparent drift also requires further, more in-depth research. Such research should identify, in the first place, the main factors underlying this surprising trend. The causes of the latter potentially might include the impact of changing research topic as well as work conditions for the returning scholar, the length of time needed for his/her re-positioning in international networks of research or, again, the not as-high-as-expected efficiency of pre-grant peer reviews.

## Conclusions

In brief, large-scale grant schemes such as the MP are indeed able to enrich the scientific landscape of a country. The MP winners were already the best, and performance further increased by 23–52% during the grant period. In particular, group leaders with a dynamic pre-grant publication track record were able to attain the most additional momentum. It is, however, still an open question as to what extent the MP or a similar grant scheme also involving international mobility is able to appeal to the best researchers determined to establish their new independent research groups in a different country. Our study may be considered an initial effort to shed some light on the ways that grant selection processes can be made more efficient in the future.
